# A Lightweight and Small Sample Bearing Fault Diagnosis Algorithm Based on Probabilistic Decoupling Knowledge Distillation and Meta-Learning

**DOI:** 10.3390/s24248157

**Published:** 2024-12-20

**Authors:** Hao Luo, Tongli Ren, Ying Zhang, Li Zhang

**Affiliations:** College of Information, Liaoning University, Shenyang 110036, China; luohao8711@163.com (H.L.); 4032342472@smail.lnu.edu.cn (T.R.); 15845866814@163.com (Y.Z.)

**Keywords:** fault diagnosis, small sample, meta-learning, knowledge distillation, lightweight

## Abstract

Rolling bearings play a crucial role in industrial equipment, and their failure is highly likely to cause a series of serious consequences. Traditional deep learning-based bearing fault diagnosis algorithms rely on large amounts of training data; training and inference processes consume significant computational resources. Thus, developing a lightweight and suitable fault diagnosis algorithm for small samples is particularly crucial. In this paper, we propose a bearing fault diagnosis algorithm based on probabilistic decoupling knowledge distillation and meta-learning (MIX-MPDKD). This algorithm is lightweight and deployable, performing well in small sample scenarios and effectively solving the deployment problem of large networks in resource-constrained environments. Firstly, our model utilizes the Model-Agnostic Meta-Learning algorithm to initialize the parameters of the teacher model and conduct efficient training. Subsequently, by employing the proposed probability-based decoupled knowledge distillation approach, the outstanding performance of the teacher model was imparted to the student model, enabling the student model to converge rapidly in the context of a small sample size. Finally, the Paderborn University dataset was used for meta-training, while the bearing dataset from Case Western Reserve University, along with our laboratory dataset, was used to validate the results. The experimental results demonstrate that the algorithm achieved satisfactory accuracy performance.

## 1. Introduction

Rolling bearings, as key components of rotating machinery in the industrial field, have a wide range of applications in various fields such as automobiles, airplanes, industrial machinery, electric motors, and home appliances [1,2,3]. Their main function is to support rotating components, reduce the friction coefficient, and improve equipment operating efficiency and reliability. However, in complex working conditions and harsh environments, rolling bearings are prone to damage, affecting the overall performance of the equipment. According to statistics from authoritative institutions, bearing failures [4] account for approximately 45% to 70% of all mechanical failures [5]. It is evident that diagnosing bearing faults is crucial. This not only allows for monitoring the real-time operational status of equipment, but also helps in the timely replacement of bearings when faults occur, thereby avoiding more serious losses and ensuring the safe and stable operation of industrial production [6].

In the early stages, fault diagnosis relied primarily on manual observation and simple tools. With technological advancements, methods utilizing vibration signals, infrared thermal signals, acoustic signals, and others emerged. The fault diagnosis method based on current vibration signals has been widely applied; however, current signals often contain a large amount of redundant information. Signal compression, as an efficient preprocessing technique, can reduce data dimensionality and computational complexity while preserving key features. For instance, Han et al. [7] proposed a fault diagnosis method for high-speed train bearings based on compressed sensing and acoustic emission, which optimizes signal reconstruction through adaptive stepsize forward–backward pursuit and dynamic thresholding. Kim et al. [8] proposed a bearing fault diagnosis technique based on convolutional neural networks and acoustic emission. Hou et al. [9] focused on high-speed train wheelset bearings and introduced an improved fingerprint feature recognition method based on acoustic emission, overcoming the limitations of existing diagnostic approaches.

With the development of computers and the rise in deep learning algorithms, intelligent diagnostic methods based on machine learning and deep learning have received widespread attention in recent years. Machine learning methods, such as Support Vector Machines (SVMs) [10] and Random Forest [11], have been widely used for fault diagnosis. Subsequently, more advanced deep learning techniques, such as convolutional neural networks (CNNs) [12] and recurrent neural networks (RNNs) [13], were proposed, which significantly enhanced diagnostic accuracy under complex working conditions. Deng et al. [14] proposed a multi-scale residual convolutional network (MRNet) for diagnosing rolling bearing faults in noisy environments. Saufi et al. [15] proposed a one-dimensional optimized convolutional neural network method (DE-1D-CNN), which integrates acoustic emission and vibration sensor signals. These signals are then input into a differential evolution algorithm to optimize hyperparameters. Kumar et al. [16] introduced a bearing fault diagnosis model based on multi-wide kernel convolutional neural networks. By using wide kernels in the convolutional layer, the model can capture a wider range of patterns, obtain both local and global features, and improve its ability to distinguish between healthy and faulty bearings.

With the development of deep learning and neural networks, model parameters are also increasing. To address the challenges posed by excessively large models, various model compression methods have been introduced. The mainstream model compression methods now include model pruning [17], model quantization [10], low rank decomposition [18], knowledge distillation [19], among others. Cheng et al. [20] introduced a hierarchical structure pruning multi-scale feature fusion residual network (HSP-MFFRN) for intelligent fault diagnosis (IFD), which effectively reduces the model size while maintaining superior diagnostic performance through multi-scale feature extraction, fusion, compression, and hierarchical structure pruning. A lightweight bearing fault diagnosis model, BearingPGA-Net, was developed by Liao et al. [21], which achieves high-speed and low-power industrial deployment through the decoupling of knowledge distillation training and the design of FPGA acceleration schemes. Lu et al. [22] designed a lightweight transfer learning framework for rolling bearings based on knowledge distillation. This framework uses a deep teacher–student model to learn domain-invariant features, combined with temperature–factor knowledge distillation and multi-core domain-adaptive methods, achieving cross-device fault diagnosis while effectively balancing computational resources and diagnostic accuracy. Dubey et al. [23] presented an automated method for VNCMD mode quantity and instantaneous frequency initialization based on amplitude spectrum scale–space representation, which was applied to bearing fault detection. The DSADRSViT-IIRL diagnostic framework for small and imbalanced datasets was developed by Gu et al. [24], integrating dual-stream adaptive deep residual shrinkage blocks and inter-class intra-class rebalancing losses to address the challenges of limited samples and class imbalance. Zhong et al. [25] introduced a method called Small Sample Dense Teacher-Assisted Knowledge Distillation (SS-DTAKD), which combines knowledge distillation and Generative Adversarial Network (GAN) techniques to tackle the challenges of large storage requirements and difficulty in obtaining sufficiently reliable training data. Traditional knowledge distillation focuses on the probability distribution over the geometric relationships of feature vectors, which weakens its ability to learn feature space structure. Therefore, probability-based knowledge distillation was proposed to capture the local structure and geometric information of feature space (i.e., feature representation spatial angle relationships) through cosine similarity of feature vectors, thus benefiting deep-level feature representation learning.

At present, significant advances have been made in small sample training methods, including data augmentation [26] and meta-learning [27] among others. Meta-learning, which provides sufficient training data to compensate for the scarcity of actual data, has gained significant attention in recent years. Model-Agnostic Meta-Learning (MAML) is a typical example of meta-learning [28], with the goal of initializing model parameters to enable good performance on new tasks using limited training data and few gradient updates. Liu et al. [29]. proposed a new continuous learning method, CL-DMAML, based on downsampling and model-agnostic meta-learning. This method addresses the challenges of incremental learning and sample imbalance in rolling bearing fault diagnosis. Abdullah et al. [30] introduced a two-level sorting meta-learning method (SSML), which uses MAML to address insufficient fault data and cross-dataset issues. This method ensures the accuracy of intelligent fault detection models and achieves second-level sorting and cross-dataset diagnosis. Lin et al. [31] designed Generalized Model Unknown Meta-Learning (GMAML) to address the limitations of homogeneous signal analysis and the challenge of extracting general diagnostic knowledge in small-sample, cross-domain bearing fault diagnosis.

However, in situations where data are scarce, small sample learning methods can effectively use limited data for training, reducing the cost of large-scale data collection and processing. In the research mentioned above, numerous scholars have already demonstrated the model-agnostic property of MAML and its superiority in the field of small-sample learning. However, few knowledge distillation methods currently address small-sample fault identification. To address these challenges, MIX-MPDKD is proposed, a method that combines knowledge distillation with MAML. First, we performed pseudo-training on the teacher model to obtain initialization parameters. Next, the trained teacher model distilled knowledge into the student model, enabling it to learn small sample recognition from the teacher. Finally, we obtained the classification model. In summary, the main contributions of this article are as follows:

1. The MAML algorithm is proposed to address the small sample problem, with the model being initialized to promote rapid convergence and effectively prevent overfitting;
2. A lightweight classification model is constructed based on the knowledge distillation algorithm, and the number of parameters is further reduced through depthwise separable convolution;
3. A probability-based decoupling knowledge distillation algorithm is proposed. In this algorithm, the probability distributions of the teacher and student models in the feature space are matched, while distinguishing between target knowledge and non-target knowledge, enabling the student model to efficiently learn the knowledge from the teacher model.

The structure of this article is outlined as follows: Section 2 provides a concise overview of the fundamental theoretical concepts underlying fault diagnosis models. Section 3 presents a detailed explanation of the proposed algorithm. Section 4 demonstrates the algorithm’s effectiveness through validation on the CWRU and laboratory bearing datasets. Lastly, Section 5 concludes the study with key findings and insights.

## 2. Background and Related Works

### 2.1. Knowledge Distillation

**Figure 1:**
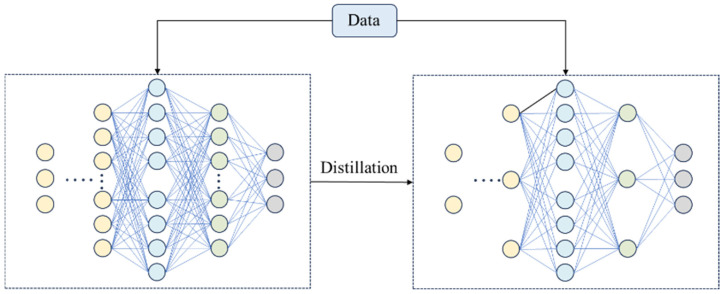
Knowledge distillation.

As shown in Figure 1, knowledge distillation [19] is a model compression technique in which knowledge is transferred from large, complex teacher models (which have good performance and intricate structures) to simpler student models. By allowing the student model to learn both the soft labels (which include inter-class relationship information) and the hard labels (i.e., the true classes) produced by the teacher model, the student model can achieve good performance even with a smaller number of parameters.

This process enables model lightweighting, reduces computational costs, and improves inference speed, making it highly significant in resource-constrained scenarios. In the process of knowledge distillation, the teacher model is first trained to achieve high accuracy and is then used to generate soft labels (i.e., the probability distribution of the model’s output). These soft labels contain rich category information and inter-category correlations, providing more information compared to traditional hard labels (i.e., true category labels). Subsequently, the student model is trained using both these soft labels and the true category labels. By minimizing the difference between the student model’s output and the teacher model’s soft labels, the student model can acquire knowledge from the teacher model and achieve model compression while maintaining high performance.

### 2.2. Depthwise Separable Convolution

Depthwise separable convolution [32] is a specialized operation in convolutional neural networks (CNNs) designed to significantly reduce computational complexity without compromising performance. This technique splits the standard convolution process into two distinct steps: depthwise convolution [33], which performs spatial filtering, and pointwise convolution [34], which handles channel mixing. Due to its efficiency, it has become a cornerstone in various lightweight neural network architectures. The parameters and computational complexity of standard convolution are defined by Equations (1) and (2), respectively, whereas those for depthwise separable convolution are outlined in Equations (3) and (4).

(1) $$D_{K}\times D_{K}\times M\times N,$$

(2) $$D_{F}\times D_{F}\times D_{K}\times D_{K}\times M\times N,$$

(3) $$D_{K}\times D_{K}\times M+1\times 1\times M\times N,$$

(4) $$D_{F}\times D_{F}\times M\times D_{K}\times D_{K}+D_{F}\times D_{F}\times M\times N,$$

In the formula, M represents the number of input channels, N denotes the number of output channels, $D_{F}\times D_{F}$ refer to the dimensions of the input feature map, and $D_{K}\times D_{K}$ corresponds to the size of the convolution kernel. The ratio of the number of parameters and the computational complexity of depthwise separable convolution compared to traditional convolution methods is given by Equations (5) and (6):

(5) $$\frac{D_{K}\times D_{K}\times M+1\times 1\times M\times N}{D_{K}\times D_{K}\times M\times N}=\frac{D_{K}\times D_{K}+N}{D_{K}\times D_{K}\times N},$$

(6) $$\begin{array}{l} \frac{D_{F}\times D_{F}\times M\times D_{K}\times D_{K}\times M+D_{F}\times D_{F}\times M\times N}{D_{F}\times D_{F}\times D_{K}\times D_{K}\times M\times N}\\ =\frac{1}{D_{K}^{2}}+\frac{1}{N}. \end{array}$$

From the above equation, it can be seen that as the size and number of convolution kernel increase, the reduction in computational complexity becomes more significant. Evidently, depthwise separable convolution is instrumental in significantly reducing both the number of parameters and the computational complexity of the model, thus providing strong support for the lightweight and efficient computation of the model.

## 3. The Proposed Algorithm

**Figure 2:**
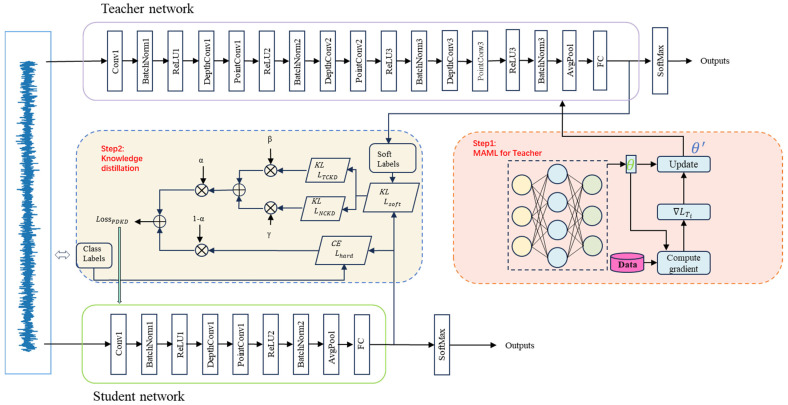
The proposed MIX-MPDKD algorithm.

The overall framework of MIX-MPDKD is illustrated in Figure 2. Firstly, vibration signals from the bearing are collected using the accelerometers installed on it. These signals are transmitted via a data acquisition card to a computer, forming the raw time-domain data. The time-domain signals are then used as inputs for diagnostic analysis with the proposed algorithm. MIX-MPDKD enhances the teacher network’s generalization ability by combining MAML with MIXCNN. Subsequently, quantization and the enhanced PDKD are iteratively applied to produce a more compact yet highly accurate student network.

### 3.1. MIXCNN

For the MIXCNN model constructed, a wide-kernel convolution [35] filter was first used to extract short-term features from the original vibration signal. After the convolution operation, ReLU was applied as the activation function, and the output values were normalized through batch normalization (BN). This process was designed to overcome the gradient dispersion problem and to accelerate network computation and training. The mathematical expression for this process is shown in Equation (7).

(7) $$y=BN(\sigma_{relu}\{\sum_{m}^{M}W_{\left(m,k\right)}*x_{\left(i+m,k\right)}\}).$$

where W is the weight matrix, x is the input data, the number of convolution kernels is k, the width of the convolution kernels is m, $\sigma_{relu}$ represents the ReLU activation function, ∗ represents the convolution operation, and y is the transformed output data.

Suppose the input for layer n is $x^{l}=(x^{l(i,1)},x^{l(i,2)},x^{l(i,3)},\ldots ,x^{l(i,j)},\ldots ,x^{l(i,p)}).$ For the BN process, first calculate the mean $\mu_{B}$, and then calculate the variance $\sigma_{B}^{2}$. After completing these two steps of calculation, standardize the data. Afterwards, learnable parameters are introduced to perform scaling and translation operations, and the final output is shown in Formulas (8)–(10).

(8) $$\mu_{B}=\frac{1}{m}\sum_{i=1}^{m}x^{l(i)},$$

(9) $$\sigma_{B}^{2}=\frac{1}{m}\sum_{i=1}^{m}{\left(x^{l(i)}-\mu_{B}\right)}^{2},$$

(10) $$y=\gamma {\hat{x}}^{l(i)}+\beta ,$$

Here, m represents the batch size, $\mu_{B}$ denotes the mean value of the batch data, and $\sigma_{B}^{2}$ signifies the variance of the batch data. The parameters γ and β are trainable variables. Depthwise separable convolution is composed of two stages: depthwise convolution and pointwise convolution. The depthwise convolution operates independently on each feature map within the individual channels, capturing spatial location-specific features. Subsequently, the processed channels are recombined, ensuring that the number of output feature maps matches the input layer’s channel count, as expressed in Equation (11).

After completing the depthwise convolution, in order to reduce information loss and prevent the vanishing gradient problem, a residual structure is used. The output of the depthwise convolution layer is added to the original input data, and the resulting sum is then processed by the pointwise convolution layer.

(11) $$z=BN(\sigma_{relu}\{\sum_{m}^{M}W_{M}*y_{i+m}\}).$$

Pointwise convolution mainly uses 1 × 1 convolution kernels to perform channel operations, which have two main functions. On the one hand, it can integrate the features of each channel after depthwise convolution, allowing for sufficient communication of information between channels; on the other hand, it can flexibly adjust the number of channels to meet the task requirements of different layers. This complete process can be represented by Equations (12) and (13):

(12) $$\hat{z}=y+z,$$

(13) $$\hat{y}=BN(\sigma_{relu}\{\sum_{k}^{K}W_{k}*\hat{z}\}).$$

**Figure 3:**
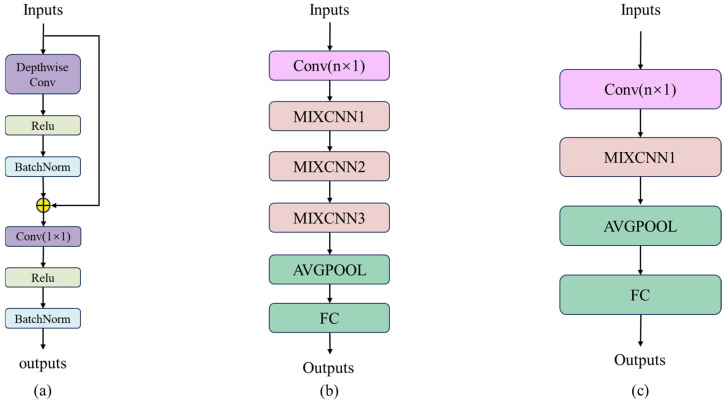
(**a**) MIXCNN. (**b**) Teacher model. (**c**) Student model.

Subsequently, the teacher and student networks are built based on MIXCNN. The specific network structure is shown in Figure 3.

After analyzing the test results of convolution kernel size and network layers, it was found that the network layers of the teacher model should not be set too deep. Based on past experience, although increasing the number of network layers may improve the accuracy of the model, this process consumes a significant amount of computational resources. More network layers can enable models to learn complex functional relationships, but they can also lead to problems. Firstly, overfitting of training data may cause the model to perform worse on new data; secondly, it is easy to cause gradient explosion or disappearance, which is not conducive to training and optimization.

This paper uses a four-layer MIXCNN convolutional network to construct the teacher model architecture and a two-layer MIXCNN convolutional network for the student model. In this architecture, the kernel size used for both wide convolution and depthwise separable convolution is 64 × 1.

### 3.2. Pseudo-Trained Teacher Model

The MAML algorithm [28] represents a widely adopted meta-learning framework that leverages auxiliary datasets, termed pseudo-training sets [29], to initialize model parameters, thereby enabling efficient adaptation to specific tasks. MAML learns a generalized parameter initialization by training across multiple related tasks, enabling the model to quickly adapt to new tasks. On unseen data, MAML generalizes by leveraging shared feature patterns across tasks and fine-tuning with a small amount of data. Under different operating conditions, the model can learn a more robust initialization through task diversity during meta-training, allowing it to adapt to new environmental changes or shifts in input distributions. Specifically, the execution process of the MAML algorithm is as follows:

Firstly, the algorithm randomly samples multiple tasks from the task distribution p(T), each task containing a small dataset. The pseudo training sets obtained by sampling are labeled as $P_{1},P_{2},P_{3},\ldots ,P_{n},$ where ach pseudo training set $P_{n}$ is represented as: $P_{n}=\{(X_{i}^{1},Y_{i}^{1}),\ldots ,(X_{i}^{N},Y_{i}^{N}),\ldots ,(X_{j}^{1},Y_{j}^{1}),\ldots ,(X_{j}^{N},Y_{j}^{N})\}$, where *i* and *j* represent different types of faults, $X_{i}$ and $Y_{i}$ represent data samples and their corresponding labels, and *N* is the number of data samples for each fault type. While extracting the pseudo training set, the MAML algorithm also selects the corresponding pseudo test sets ${\hat{P}}_{1},{\hat{P}}_{2},{\hat{P}}_{3},\ldots ,{\hat{P}}_{n}$. These pseudo-test sets and pseudo-training sets work together to provide a basis for the training and evaluation of the model.

Subsequently, multiple pseudo-training sets are employed to refine the model’s initial parameters, ensuring rapid convergence across a variety of scenarios. MAML first performs an inner loop update on the pseudo training set. For each task $T_{i}$, initialize the model using the current model parameters θ and calculate the loss $L_{T_{i}}(f_{\theta })$; the loss function uses cross entropy loss. Update the model parameters using gradient descent to obtain task specific parameters; the specific calculation formula is shown in Equation (14):

(14) $$\theta_{i}^{\prime }=\theta -\alpha \nabla_{\theta }L_{T_{i}}(f_{\theta }).$$

where *α* is the pseudo learning rate. After completing the inner loop update of each task, MAML enters the outer loop update, which calculates the meta loss of the model based on the update parameter ${\theta^{\prime }}_{i}$ prime of each task $L_{\mathrm{meta}}$; the specific calculation formula is shown in Equation (15):

(15) $$L_{meta}=\sum_{i=1}^{n}\left(f_{\theta_{i}^{\prime }}\right).$$

where $L_{\mathrm{meta}}$ is the cross-entropy loss after training on the *i*th pseudo training set. Finally, borrowing $L_{\mathrm{meta}}$ updates the initial parameters of the model to achieve initialization, as shown in Equation (16):

(16) $$\theta \leftarrow \theta -\beta \nabla_{\theta }L_{meta}.$$

where β is the learning rate for updating model parameters. After multiple updates, the model can adapt to a variety of different situations, and even in situations with limited data, it can quickly converge, thereby achieving accurate classification under small sample conditions.

### 3.3. Proposed Knowledge Distillation Based on Probability Decoupling

Knowledge distillation (KD) and its variants often transfer knowledge through the output classification layer of the network, while probabilistic knowledge distillation (PKD) methods transfer knowledge by matching the probability distributions between the teacher and student models in the feature space. PKD captures the geometric structure of the feature space by constructing conditional probability distributions among the samples and models the affinity between the samples as probability distributions (PD). This method does not require strict alignment of model hierarchy or dimensions. It improves the generalization ability of student models by maintaining mutual information, making it suitable for various tasks, such as cross-modal learning. In this study, in order to improve the quality and efficiency of KD, both the PD and category labels of the samples are considered. The former maintained the geometric relationship of the teacher feature space in the low dimensional space of the student network, while the latter enhanced the mapping relationship between the classification labels and other transfer set data.

Firstly, to effectively capture local structural information in the feature space, we constructed conditional probability distributions between the samples within the feature space. This conditional probability distribution indicates the probability of each sample selecting its neighbors, which can more accurately characterize the local morphology between samples. For teacher networks and student networks, their respective conditional probability distributions are defined by Equations (17) and (18), respectively.

(17) $$p_{T,i|j}=\frac{K(y_{T,i},y_{T,j})}{\sum_{k=1,k\neq i}^{N}K(y_{T,i},y_{T,j})},$$

(18) $$p_{S,i|j}=\frac{K(y_{S,i},y_{S,j})}{\sum_{k=1,k\neq i}^{N}K(y_{S,i},y_{S,j})}.$$

where K(⋅) is the kernel function, and the cosine kernel function [36] is selected. The formula is shown in Equation (19):

(19) $$K_{cosine(a,b)}=\frac{1}{2}\left(\frac{a^{T}b}{∥a{∥}_{2}∥b{∥}_{2}}+1\right)\in [0,1].$$

where α and b are the inputs of the teacher or student network. The cosine kernel function is sensitive to vector direction and unaffected by vector length, making it effective for measuring data similarity.

Let $Q=\{q(x_{1}),q(x_{2}),\ldots ,q(x_{N})\}$ represent the class labels of the migration set x. The teacher network initially undergoes training using hard labels, and its output, after being processed through a softmax function, is then utilized as soft labels to guide the training of the student network. During the training of the teacher network, its loss function $L_{T}(Q,P_{T})$ is defined as the cross-entropy between the output probability distribution (PD) and the category label, as given in Equation (20).

(20) $$L_{T}(Q,P_{T})=-\frac{1}{N}\sum_{i=1}^{N}q(x_{i})log(p_{T}(x_{i})).$$

For student networks, hard labels provide clear classification objectives, giving the student model a clear direction for optimization and serving as a reliable initial learning basis to quickly establish basic discriminative abilities. Therefore, the loss function of the student model $L_{S}$ has both $L_{hard}$ and $L_{soft}$, as shown in Equation (21) below.

(21) $$L_{S}(Q,P_{T},P_{S})=(1-\alpha )\cdot L_{hard}(Q,P_{S})+\alpha \cdot L_{soft}(P_{T},P_{S}).$$

where α is the weighting coefficient that balances the two loss functions. The setting of α is not completely the same under different experimental purposes and conditions. According to experimental results, α = 0.2 may better balance the two losses, which will be discussed in the next section.

The loss function $L_{hard}(Q,P_{S})$ is characterized as the cross-entropy between the category labels and the probability distribution (PD) produced by the student network, as shown in Equation (22).

(22) $$L_{hard}(Q,P_{S})=-\sum_{i=1}^{N}q(x_{i})log(p_{S}(x_{i})).$$

The loss $L_{soft}(P_{T},P_{S})$ is calculated using KL divergence [37] to approximate the PD between the teacher–student network, expressed as (23).

(23) $$L_{soft}(P_{T},P_{S})=-\sum_{i=1}^{N}p_{T}(x_{i})log\frac{p_{T}(x_{i})}{p_{S}(x_{i})}.$$

However, using $L_{S}\left(Q,P_{T},P_{S}\right)$ as the loss function results in the complete transfer of the teacher’s logic to the student under the same guidance. The student model, however, should possess the ability to filter the knowledge it receives, enhancing information pertinent to the current target class while downplaying irrelevant knowledge. This coupling effect diminishes both the effectiveness and flexibility of the model across different tasks. To address this, we introduce a probability-based decoupling knowledge distillation approach, in which $L_{S}\left(Q,P_{T},P_{S}\right)$ is decomposed into a weighted sum of two components: the target class and the non-target class [38].

Let $p_{t}$ represent the probability of the target class and $p_{/t}$ denote the probability of the non-target class. Consequently, we derive Equations (24) and (25):

(24) $$p_{t}=\frac{exp(z_{t})}{\sum_{j=1}^{C}exp(z_{j})},p_{/t}=\frac{\sum_{i=1,i\neq t}^{C}exp(z_{t})}{\sum_{j=1}^{C}exp(z_{j})}.$$

(25) $$\hat{p}=\frac{p_{t}}{p_{/t}}=\frac{exp(z_{t})}{\sum_{i=1,i\neq t}^{C}exp(z_{i})}.$$

Then, $L_{soft}(P_{T},P_{S})$ can be decomposed into Equation (26):

(26) $$\begin{array}{l} L_{soft}\left(P_{T},P_{S}\right)\\ =-p_{t}^{T}log\frac{p_{t}^{S}}{p_{t}^{T}}-\sum_{i=1,i\neq t}^{C}p_{i}^{T}log\frac{p_{i}^{S}}{p_{i}^{T}}\\ =\underset{L_{soft}^{TCKD}}{\underset{︸}{-p_{t}^{T}log\frac{p_{t}^{S}}{p_{t}^{T}}-p_{/t}^{T}log\frac{p_{/t}^{S}}{p_{/t}^{T}}}}-\underset{L_{soft}^{NCKD}}{\underset{︸}{p_{/t}^{T}\sum_{i=1,i\neq t}^{C}{\hat{p}}_{i}^{T}log\frac{{\hat{p}}_{i}^{S}}{{\hat{p}}_{i}^{T}}}} \end{array}$$

where $L_{soft}^{TCKD}$ represents the KL loss between the probabilities of the target class teachers and students, which is called Target Class Knowledge Distillation (TCKD); $L_{soft}^{NCKD}$ represents the KL loss between non-target class teacher–student probabilities, known as Non-Target Class Knowledge Distillation (NCKD) [21].

Finally, the obtained $L_{PDKD}$ as shown in Equation (27) is as follows:

(27) $$L_{PDKD}(Q,P_{T},P_{S})=(1-\alpha )L_{hard}(Q,P_{S})+\alpha (\beta L_{soft}^{TCKD}+\gamma L_{soft}^{NCKD}).$$

In this context, $L_{soft}^{TCKD}$ and $L_{soft}^{NCKD}$ are weighted by two hyperparameters, β and γ, to balance the contributions of each term. In bearing fault diagnosis, prioritizing TCKD while minimizing NCKD (β > γ) typically results in improved performance. By optimizing this decoupling loss, the knowledge acquired by the teacher model is more effectively transferred to the student model, thereby enhancing the performance of the student network.

## 4. Experiments and Analysis of Results

### 4.1. Experimental Details

This article evaluates the MIX-MPDKD model using the CWRU dataset [39] and the laboratory dataset, initializing the teacher network with the Paderborn University (PU) dataset [40] as a pseudo-training set. We compare the effectiveness of our algorithm with three classic lightweight models: the Wide Depth Convolutional Neural Network (WDCNN) [41], the Residual Neural Network (Resnet) [42], and the KD-ResNet-DA Network [22]. The parameters, FLOPs, and inference time of the comparison algorithm and the latest lightweight algorithm are shown in Table 1. In the implementation process, all the algorithms were encoded using PyTorch 2.0 with Python 3.11 and run on an NVIDIA GeForce RTX 4070Ti GPU to ensure consistency and reproducibility of the experimental environment.

For all the models in the comparison, we used the stochastic gradient descent (SGD) as the optimizer, and the training epoch was set to 50. Based on the experimental results of 4.3.3, we set α to 0.2. A grid search algorithm was then employed to determine the optimal values for β and γ. According to Equation (26), β should be greater than γ. Therefore, the search range for β was [0.5, 1, 2, 5, 8], and for γ was [0.1, 0.2, 0.5, 1, 2]. Based on the results in Table 2, the final values were β = 5 and γ = 0.1.

Both the CWRU and laboratory datasets contained 30 training samples, each with 2048 data points. A total of 2048 segmentation starting points were randomly selected using encoding, with a stride of 28 to resample the original signal. The training and validation sets were split in a 2:1 ratio, with 100 test samples.

We benchmarked the performance of our algorithm using commonly used metrics: accuracy and the F1 score, which are standard in bearing fault diagnosis. The definitions of these two metrics are provided in Equations (28) and (29):

(28) $$Accuracy=\frac{TP+TN}{TP+FN+FP+TN},$$

(29) $$F1score=\frac{2\cdot TP}{2\cdot TP+FP+FN}.$$

### 4.2. Data Descriptions

#### 4.2.1. PU Dataset

The PU dataset [39] was collected by the Paderborn University Bearing Data Center. It contained various fault vibration and current signals from 32 bearings. The bearings in this dataset were divided into three groups: the first group consisted of six healthy bearings; the second group included 12 artificially damaged bearings, which were further categorized into 7 with outer ring faults and 5 with inner ring faults; the third group contained 14 bearings damaged due to accelerated life testing, including 5 with outer ring faults, 6 with inner ring faults, and 3 with multiple faults. This dataset collected data under varying speeds, load torques, and radial forces, where the combination of these conditions altered the fault characteristics to reflect real-world complexity. It includes a variety of single and compound faults, as well as multi-point faults with varying degrees of damage, highlighting the complexity and variability of bearing fault data. In this study, both real and artificially damaged bearings were used for pseudo-training of the teacher model. Among the artificially damaged bearings, two healthy bearings, four types of inner ring faulty bearings, and four types of outer ring faulty bearings were selected; among the naturally damaged bearings, one healthy bearing, three inner ring faulty bearings, three outer ring faulty bearings, and three bearings with multiple faults were selected. The PU dataset is well-regarded in mechanical fault diagnosis, with diverse operating conditions and comprehensive annotations. It helps models learn generalizable features for adapting to various fault diagnosis tasks. Therefore, data from artificially damaged bearings under the N15M01F10 and N15M07F10 conditions are selected for MAML training, while data from naturally damaged bearings under the N15M01F10 operating condition are selected for MAML testing.

#### 4.2.2. CWRU Dataset

**Figure 4:**
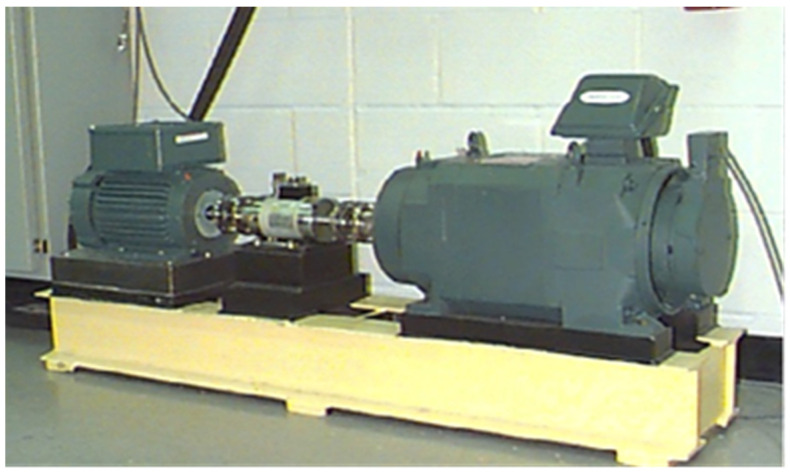
CWRU experimental device.

As shown in Figure 4, the CWRU dataset [38] is derived from a mechanical system driven by a motor. Different levels of fault severity are simulated, with single-point defects introduced on bearings at both the drive end and fan motor end, each with different diameters. Each bearing is tested under motor loads of 0, 1, 2, and 3 horsepower across four different conditions. Under these conditions, the corresponding rotational speeds are 1797, 1772, 1750, and 1730 revolutions per minute, respectively.

In this study, vibration signals from the drive end bearings were sampled at a frequency of 12 kHz under four different loads for model validation. These signals were used to construct four datasets, as shown in Table 3, labeled C0, C1, C2, and C3, corresponding to motor loads of 0, 1, 2, and 3 horsepower, respectively. Each dataset contains 10 types of bearing faults for fault classification, labeled as follows: (1) normal state (NC), i.e., no faults; (2) ball faults (BF1, BF2, BF3) with diameters of 0.007, 0.014, and 0.021 inches, respectively; (3) inner ring faults (IF1, IF2, IF3) with diameters of 0.007, 0.014, and 0.021 inches; (4) outer ring faults (OF1, OF2, OF3) with diameters of 0.007, 0.014, and 0.021 inches.

#### 4.2.3. Laboratory Dataset

**Figure 5:**
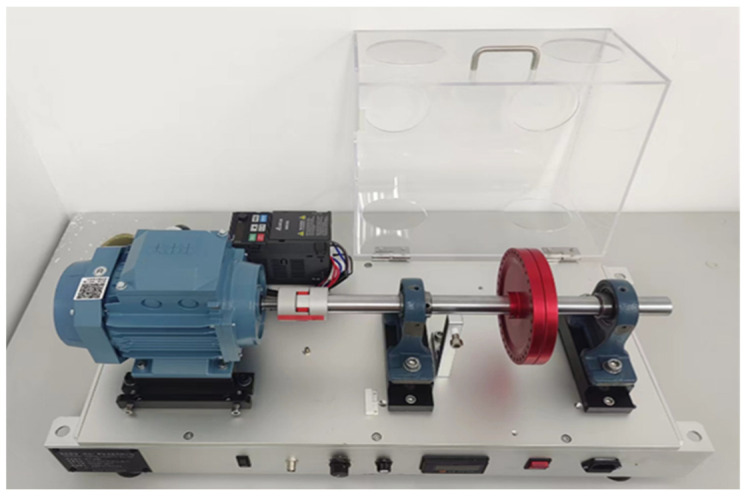
Laboratory bearing experimental device.

The laboratory bearing device consists of motors, acceleration sensors, supporting bearings, and other components, as shown in Figure 5. The collection frequency is 5 kHz, and the sensor is placed at the 12 o’clock position of the bearing to collect diagnostic signals. Data are collected at speeds of 950 rpm, 1000 rpm, and 1050 rpm under a 2 HP load condition.

As shown in Table 4, the collected data types include normal, rolling element failure, inner ring failure, and outer ring failure. Each type corresponds to three different damage diameters: 0.2 mm, 0.3 mm, and 0.4 mm. For example, at a speed of 1000 rpm, there are 9 fault states, including Light Inner Ring Fault (LIR), and a total of 10 types of data, including Normal (N). The data collected under the three operating conditions of 950 rpm, 1000 rpm, and 1050 rpm are referred to as L1, L2, and L3, respectively.

### 4.3. Experimental Results on the CWRU Dataset

#### 4.3.1. Comparative Experiment

To comprehensively evaluate the model’s resilience to noise, Gaussian white noise with different signal-to-noise ratios (SNRs) was added to the original bearing data. The equation for calculating the signal-to-noise ratio is provided in Equation (30).

(30) $$SNR=10{log}_{10}\frac{P_{signal}}{P_{noise}}.$$

$P_{signal}$ represents the power of the signal, while $P_{noise}$ represents the power of the noise.

**Figure 6:**
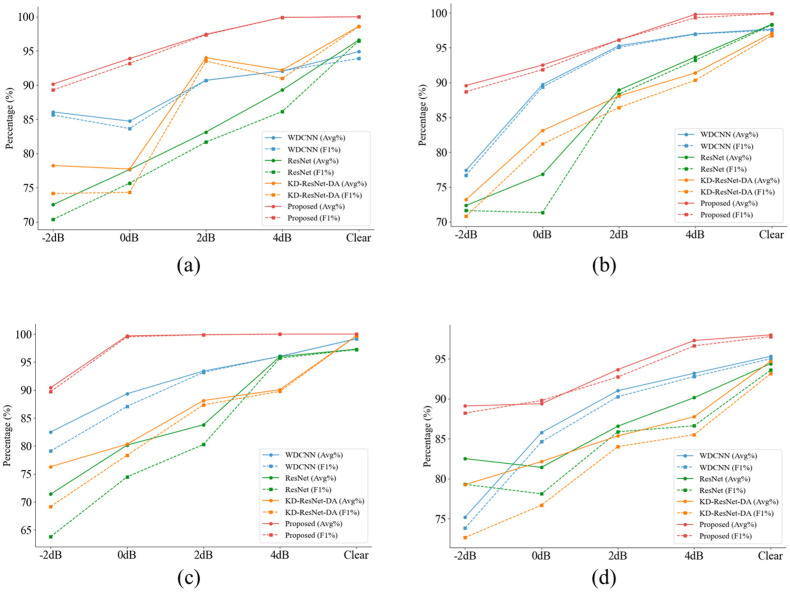
The accuracy and F1 score of CWRU dataset under different signal-to-noise ratios. (**a**) The result of C0. (**b**) The result of C1. (**c**) The result of C2. (**d**) The result of C3.

The classification results of the four models under different noise conditions in the CWRU dataset are presented in Table 5. Notably, compared to other competitive algorithms, our proposed model performs exceptionally well under various noise levels and operating conditions. Figure 6 clearly shows the differences between diagnostic algorithms and the variations in diagnostic performance of the same algorithm under different noise conditions.

Overall, under the four conditions, our proposed algorithm achieved an average accuracy of 99.48% across all four datasets. For example, on the C2 dataset with 0 dB noise, our algorithm achieved remarkable results, with an accuracy of 99.70% and an F1 score of 99.52%. In contrast, the second-best performing WDCNN algorithm achieved an accuracy of only 89.34% and an F1 score of 87.08%. These results clearly demonstrate that MIX-MPDKD is an effective algorithm under noisy conditions, consistently delivering highly competitive performance across different scenarios.

**Figure 7:**
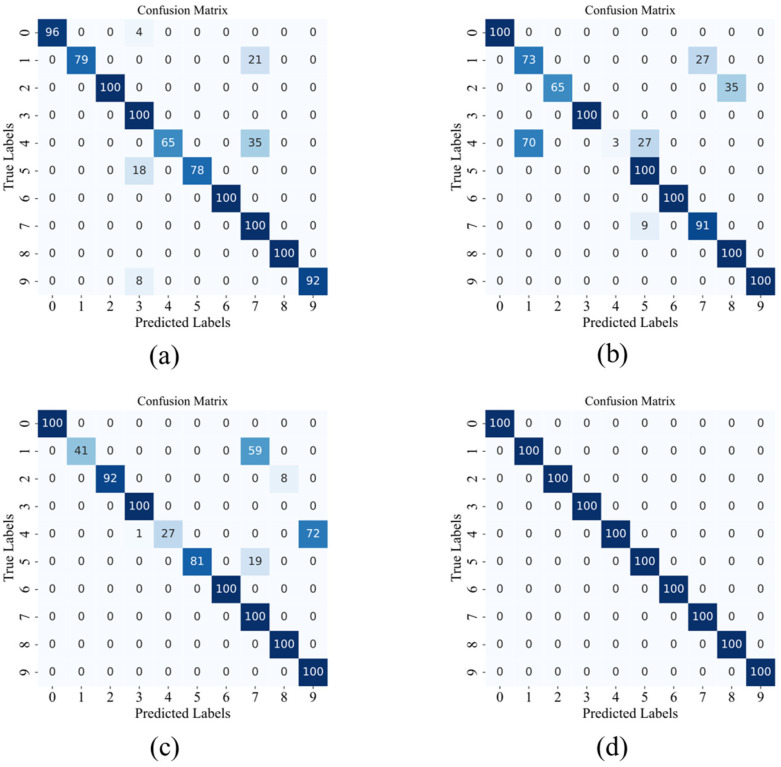
Confusion matrix of different models under 4 dB condition in C0 dataset. (**a**) WDCNN (**b**) Resnet. (**c**) KD-ResNet-DA. (**d**) Proposed.

In this study, to comprehensively evaluate the model’s performance in various classification tasks, we plotted the confusion matrix for the C0 dataset under 4 dB conditions, as shown in Figure 7. A signal-to-noise ratio of 4 dB was chosen to better reflect the model’s diagnostic performance under noisy conditions. The confusion matrix shows the predictive accuracy and error types for each class of samples. Compared to overall accuracy, it provides more detailed information on classification performance, including correct and incorrect classifications, error types, and inter-class similarity analysis. This is valuable for verifying the model’s accuracy for the target class.

**Figure 8:**
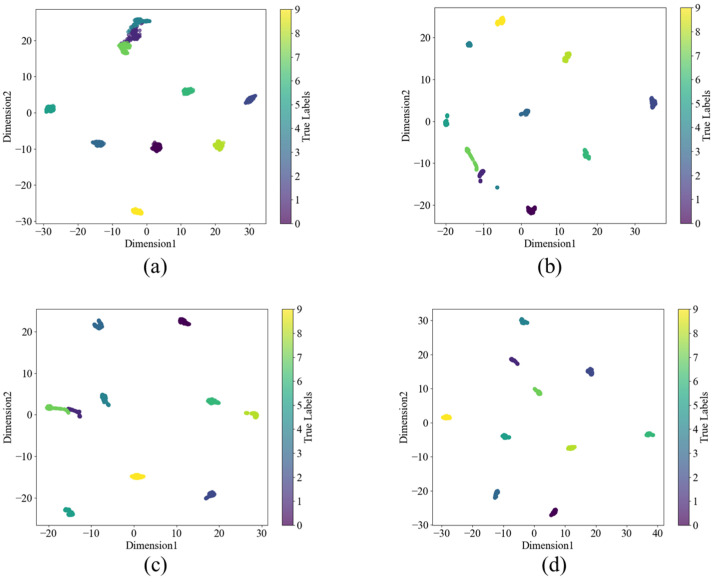
t-SNE of different models under 4 dB condition in C0 dataset (**a**) WDCNN (**b**) Resnet (**c**) KD-ResNet-DA (**d**) Proposed.

The t-SNE method maps data to a two-dimensional plane, enabling a more intuitive analysis of the model’s contribution and role. The visualization results of the output features from four different model networks are shown in Figure 8. From the figure, it can be observed that WDCNN cannot fully distinguish between different category labels, while KD-ResNet-DA can classify them, but the categories are not sufficiently compact. In the model proposed in this study, similar sample points are tightly clustered, creating better classification conditions for subsequent classifiers.

#### 4.3.2. Ablation Experiment

The ablation experiment plays a crucial role in verifying the effectiveness of the proposed method for bearing fault diagnosis. To investigate the synergy between knowledge distillation based on probability decoupling and the proposed MIXCNN architecture, we conducted ablation studies on the C1 dataset with a signal-to-noise ratio of −2 dB. In this study, we compared the following models: (1) the student model (S) with cross entropy as the loss function alone, (2) the student model (KD) that undergoes traditional distillation, (3) the student model (DKD) that undergoes ordinary decoupling knowledge distillation, and (4) the model with ordinary convolution (CNN-MPDKD) replacing the network structures of the teacher and student models with corresponding sizes. These models were compared to our proposed algorithm. On all the datasets used, noise with a signal-to-noise ratio of −2 dB was added to the vibration signals to increase the difficulty of training, so that the differences among the models could be manifested more obviously. To explore the impact of different sample quantities on the classification accuracy, in Dataset C1, the number of training samples gradually increased from 10 to 200, and the obtained diagnostic accuracy is shown in Table 6.

**Figure 9:**
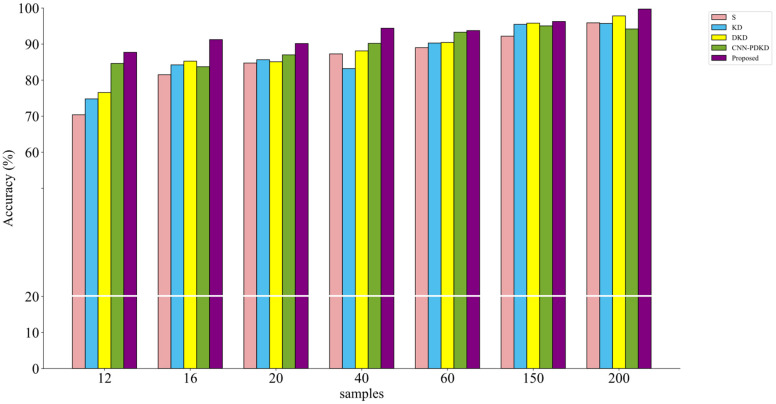
Histograms of 5 models under different noise conditions.

**Figure 10:**
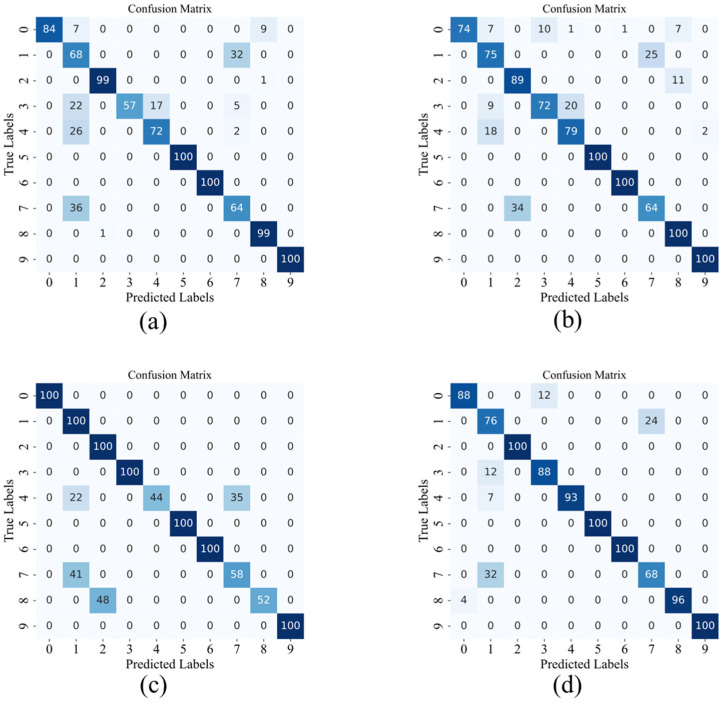
Confusion matrix of different models. (**a**) KD. (**b**) DKD. (**c**) CNN-MPDKD. (**d**) Proposed.

To compare the results between models more intuitively, we plotted the data from the table as a histogram (Figure 9) and a confusion matrix (Figure 10). Upon observation, we found that the student model trained with our proposed algorithm achieved an average accuracy improvement of 8.9% compared to the untrained student model. Compared to the student model trained with ordinary decoupling distillation, the accuracy improved by 4.8%. Additionally, compared to the CNN-MPDKD model, the average accuracy increased by 3.6%, while the number of parameters was significantly reduced. These results demonstrate that our innovation is both practical and effective.

#### 4.3.3. The Influence of Parameter α on Experimental Results

By tuning the value of α, the relative contributions of the two loss components can be effectively balanced, thereby optimizing the knowledge transfer from the teacher model to the student model. When α is close to 0, the hard loss is weighted more heavily, directing the student model to focus on the hard labels associated with fault detection tasks during training. In contrast, as α approaches 1, the soft loss becomes more prominent, encouraging the student model to prioritize the soft labels provided by the teacher. This adjustment allows the student model to generate smoother and more generalized predictions.

**Figure 11:**
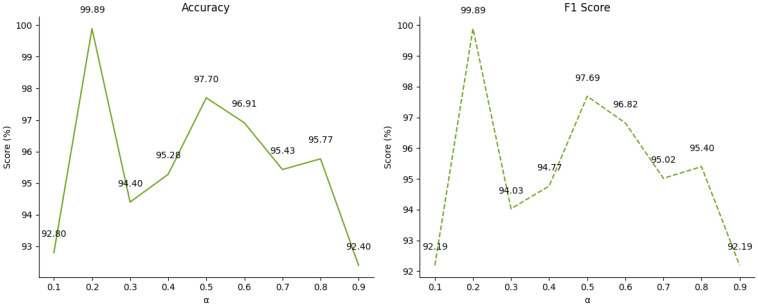
The influence of alpha on experimental accuracy and F1 score.

Figure 11 illustrates the impact of varying α values on the diagnostic performance of MIX-MPDKD. In general, the optimal choice of α depends on the specific task and dataset, as different values can produce distinct outcomes. To further explore this, we conducted experiments to examine the effect of distillation loss weights on diagnostic performance.

As shown in Figure 11 the MIX-MPDKD model achieves the highest accuracy when α is set to 0.2. However, when α increases to 0.3, there is a significant drop in accuracy, reaching its lowest point. As α approaches 0.5, performance improves markedly, though this is followed by a gradual decline. Notably, when α is reduced to 0.1, the accuracy decreases sharply. These results underscore the critical role that both classification loss and distillation loss play in enhancing the model’s overall performance.

### 4.4. Experimental Results on the Laboratory Dataset

#### 4.4.1. Comparative Experiment

Comparative experiments on the laboratory dataset were continued to be conducted by us. To ensure fairness, the hyperparameters of the models involved were coordinated and made consistent with those used in the proposed algorithm in this paper. The number of training and testing samples was the same as in the experiments comparing the CWRU dataset. Due to the significant impact of noise on the laboratory dataset, the data waveform was almost completely obscured at a signal-to-noise ratio of 0 dB. Therefore, we added Gaussian white noise with signal-to-noise ratios ranging from 0 to 6 dB to perform anti-noise experiments. The comparison results of different models are shown in Table 7.

**Figure 12:**
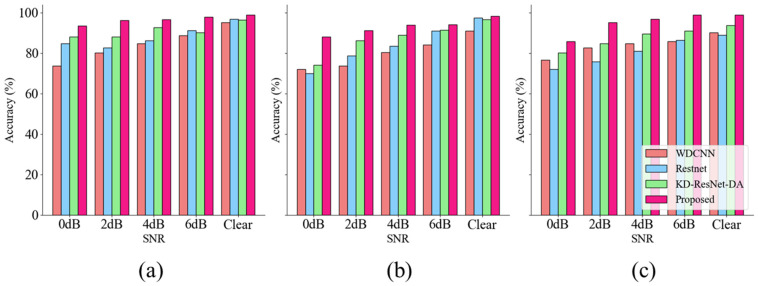
Accuracy under different noise conditions in Laboratory datasets. (**a**) L1. (**b**) L2. (**c**) L3.

**Figure 13:**
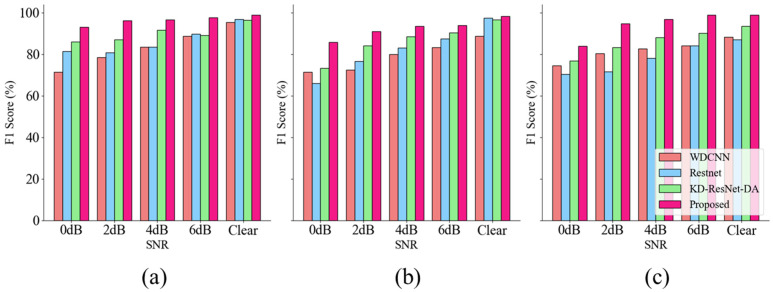
F1 scores under different noise conditions in Laboratory datasets (**a**) L1 (**b**) L2 (**c**) L3.

The results indicate that, in the presence of limited samples and noise, WDCNN and Resnet often struggle to achieve satisfactory results, while KD-ResNet-DA performs relatively better. To more intuitively observe the classification results of different models under varying noise conditions and datasets, we present the obtained accuracy and F1 scores as bar charts in Figure 12 and Figure 13. From the graphs, it can be seen that our proposed MIX-MPDKD model achieves the best performance in both accuracy and F1 score. In terms of prediction accuracy for the test samples, MIX-MPDKD achieved 98.80%, which is 2.35% higher than KD-ResNet-DA, 2.07% higher than Resnet, and 3.60% higher than WDCNN.

**Figure 14:**
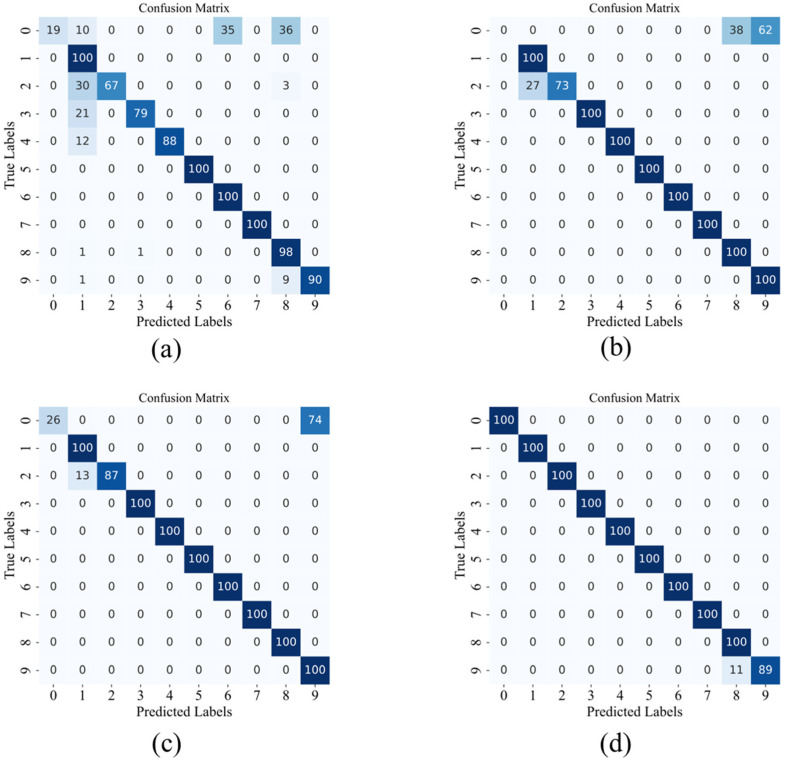
Confusion matrices of different models in the L3 dataset with a signal-to-noise ratio of 6 dB. (**a**) WDCNN. (**b**) Resnet. (**c**) KD -ResNet-DA. (**d**) Proposed.

The confusion matrix further validates the recognition ability of each model for different fault states. We have plotted the confusion matrix in Figure 14 for the L3 dataset with a signal-to-noise ratio of 6 dB. In this case, the MIX-MPDKD model achieved an accuracy of 100% for all the labels except for label 8. From Figure 14, it is clear that, under the same experimental conditions, other models exhibited a performance gap compared to our proposed model in identifying true examples.

**Figure 15:**
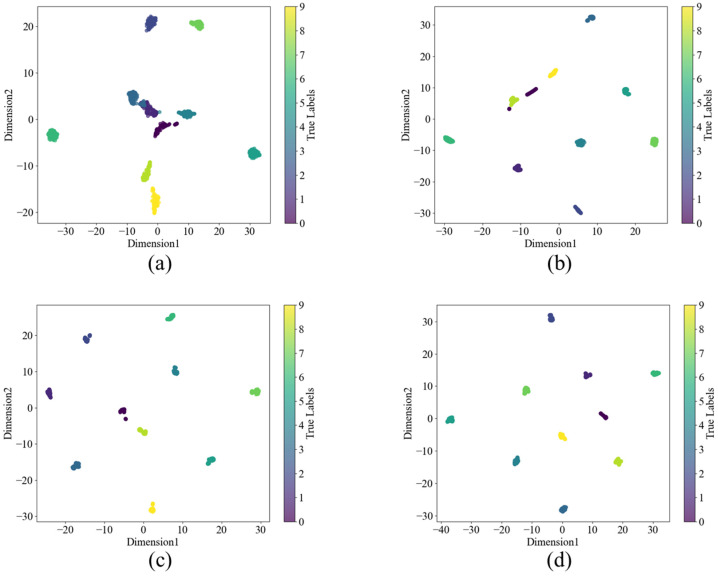
t-SNE of different models in the L3 dataset with a signal-to-noise ratio of 6 dB. (**a**) WDCNN. (**b**) Resnet. (**c**) KD -ResNet-DA. (**d**) Proposed.

Figure 15 shows the t-SNE visualization results for the L3 dataset with a signal-to-noise ratio of 6 dB. Among the models, KD-ResNet-DA achieved high scores in accuracy, the F1 score, and t-SNE visualization analysis. However, although the KD-ResNet-DA model can effectively identify various bearing health conditions, it does not integrate them effectively. For WDCNN, the classification results for faults with labels 1 and 3 are suboptimal. The results show that, under the same experimental conditions, MIX-MPDKD performs better at identifying fault types, classifying, and separating them compared to other models, and its performance surpasses that of the comparative models. This suggests that our model achieves clearer decision boundaries, clustering different fault types more effectively.

#### 4.4.2. Ablation Experiment

To further validate the effectiveness of the model improvements, we conducted ablation experiments on the laboratory dataset. The comparison models and training sample sizes are consistent with those in the CWRU dataset experiments. By examining the effectiveness and contribution of each improvement individually, we can confirm their impact on the overall model.

**Figure 16:**
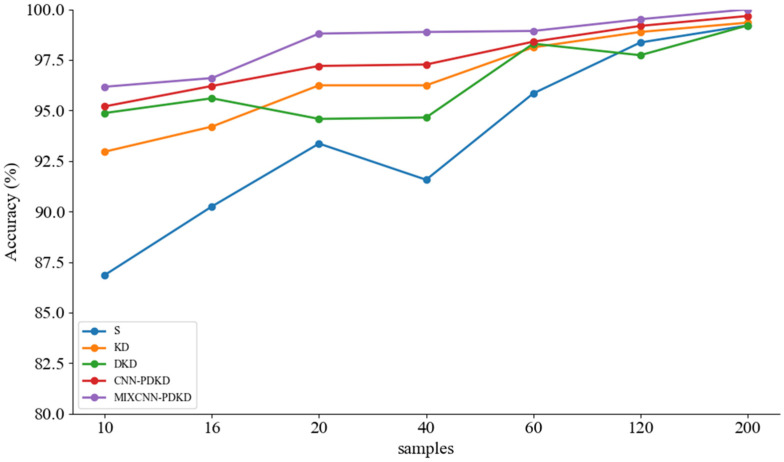
Line graph of laboratory dataset ablation experiment results.

The experiment used data from the L1 dataset without noise, and the specific results are shown in Table 8. To clearly demonstrate the performance trends of each model as the training sample size increases, and to better compare the experimental results across models, we plotted the corresponding line chart in Figure 16. From Figure 16, it can be seen that the accuracy of the CNN-PDKD model, which replaces MIXCNN with CNN, is similar to that of our model. However, our model has approximately one-twentieth of its parameter count. Our proposed model not only improves accuracy but also maintains a lightweight structure, effectively validating the effectiveness of knowledge distillation based on probability decoupling.

#### 4.4.3. Anti-Noise Experiment

To investigate the noise immunity of the proposed algorithm against various noises, 45 training samples are used. Salt-and-pepper noise with varying signal-to-noise ratios is added to the original signals in the L1 dataset. The experimental results are shown in Table 9.

## 5. Conclusions

In this article, we proposed an innovative lightweight fault diagnosis algorithm, MIX-MPDKD, to address the challenges posed by the complexity of traditional models. The algorithm incorporates three key technologies: MAML, MIXCNN, and PDKD. MAML enables the teacher model to quickly adapt to small-sample recognition and classification through pseudo-training. The design of MIXCNN reduces the number of parameters in traditional CNNs while incorporating residuals to mitigate issues such as vanishing gradients. It also captures key features of global signals, avoiding cumbersome operations. PDKD provides a novel mechanism for the student model to learn the fault recognition ability of the teacher model, offering efficient feature extraction for the entire framework. The lightweight design of MIX-MPDKD not only reduces learning parameters and computational complexity but also provides an innovative and feasible solution for fault diagnosis tasks. This algorithm highlights its unique advantages and practicality in solving small-sample fault diagnosis problems, offering a reliable solution for overcoming challenges in real-world applications.

Experimental results show that our algorithm not only features a lightweight design but also outperforms existing fault diagnosis algorithm based on CNNs and knowledge distillation in terms of robustness. This superiority is validated through experiments on the PU dataset, CWRU dataset, and laboratory data. Compared to the other algorithms discussed in this paper, the proposed distillation algorithm demonstrates significant advantages in complexity and diagnostic accuracy, making our approach a more effective and reliable solution in the field of fault diagnosis.

Although the final student model has successfully acquired the ability to diagnose with small samples, the training of the teacher model still relies on vast amounts of data. Reducing the teacher model’s reliance on data volume will be the key objective of our next stage of work.

## Figures and Tables

**Table 1:** Different algorithm parameters, FLOPs, and inference time.

Algorithms	#Parameters	#FLOPs	#Time
WDCNN	66.79 K	1.61 M	1.63 s
Resnet	25.19 K	3.27 M	1.26 s
KD-ResNet-DA	262.5 K	33.3 M	7.34 s
MPnet [43]	16.9 K	150.6 M	8.96 s
MSFsNet [44]	45 K	15.1 M	2.66 s
CNN-MPDKD	251.13 K	561.12 M	22.03 s
Proposed	13.51 K	2.74 M	1.58 s

**Table 2:** The experimental accuracy on the L1 dataset.

β	0.5	1	2	5	8
Avg%	94.56	95.11	97.36	98.80	98.46
**γ**	**0.1**	**0.2**	**0.5**	**1**	**2**
Avg%	98.77	98.01	97.55	97.24	95.34

**Table 3:** Detailed description of CWRU dataset.

Labels	Dataset				Fault Type	Fault Diameter	Train/Val/Test
	C0	C1	C2	C3			
0	0	1	2	3	Normal	0	20/10/100
1	0	1	2	3	Ball	0.007	20/10/100
2	0	1	2	3		0.014	20/10/100
3	0	1	2	3		0.021	20/10/100
4	0	1	2	3	Inner Race	0.007	20/10/100
5	0	1	2	3		0.014	20/10/100
6	0	1	2	3		0.021	20/10/100
7	0	1	2	3	Out Race	0.007	20/10/100
8	0	1	2	3		0.014	20/10/100
9	0	1	2	3		0.021	20/10/100

**Table 4:** Detailed description of laboratory dataset.

Labels	Dataset			Fault Type	Labels	Dataset			Fault Type
	L1	L2	L3			L1	L2	L3	
0	0	1	2	Normal	5	0	1	2	Inner Race (Moderate)
1	0	1	2	Ball (Minor)	6	0	1	2	Inner Race (Severe)
2	0	1	2	Ball (Moderate)	7	0	1	2	Out Race (Minor)
3	0	1	2	Ball (Severe)	8	0	1	2	Out Race (Moderate)
4	0	1	2	Inner Race (Minor)	9	0	1	2	Out Race (Severe)

**Table 5:** Accuracy and F1 score under different signal-to-noise ratio conditions in the CWRU dataset.

Dataset	Algorithm	SNR = −2 dB		SNR = 0 dB		SNR = 2 dB		SNR = 4 dB		Clear	
		Avg%	F1%	Avg%	F1%	Avg%	F1%	Avg%	F1%	Avg%	F1%
C0	WDCNN	86.08	85.64	84.74	83.66	90.71	90.71	92.08	92.09	94.90	93.89
	Resnet	72.52	70.36	77.66	75.67	83.14	81.67	89.30	86.15	96.60	96.47
	KD-ResNet-DA	78.25	74.16	77.73	74.31	94.02	93.53	92.22	90.99	98.60	98.59
	Proposed	90.16	89.31	93.89	93.19	97.44	97.37	99.92	99.92	100	100
C1	WDCNN	77.42	76.71	89.72	89.36	95.28	95.05	97.00	96.94	97.66	97.51
	Resnet	72.36	71.63	76.83	71.33	88.92	88.33	93.68	93.18	98.36	98.27
	KD-ResNet-DA	73.21	70.82	83.11	81.18	88.07	86.41	91.39	90.33	97.09	96.75
	Proposed	89.59	88.70	92.51	91.86	96.12	96.12	99.80	99.31	99.90	99.89
C2	WDCNN	82.51	79.09	89.34	87.08	93.40	93.18	96.01	96.03	99.16	99.16
	Resnet	71.4	63.8	80.17	74.49	83.80	80.28	95.98	95.72	97.30	97.24
	KD-ResNet-DA	76.30	69.19	80.33	78.36	88.13	87.36	90.07	89.77	99.69	99.69
	Proposed	90.43	89.73	99.70	99.52	99.89	99.89	100	100	100	100
C3	WDCNN	75.21	73.84	85.78	84.63	91.02	90.27	93.20	92.79	95.32	95.01
	Resnet	82.52	79.3	81.43	78.12	86.58	85.88	90.15	86.63	94.40	93.60
	KD-ResNet-DA	79.27	72.66	82.16	76.70	85.36	84.03	87.76	85.52	94.69	93.14
	Proposed	89.11	88.21	89.39	89.80	93.66	92.73	97.30	96.63	98.00	97.79

**Table 6:** The ablation experiment accuracy in the CWRU dataset.

Algorithm	10	16	20	40	60	120	200
S	70.4%	81.5%	84.75%	87.28%	89.02%	92.2%	95.9%
KD	74.8%	84.24%	85.67%	83.21%	90.3%	95.48%	95.72%
DKD	76.57%	85.27%	85.11%	88.11%	90.45%	95.82%	97.83%
CNN-MPDKD	84.63%	83.73%	87.02%	90.2%	93.3%	95.06%	94.2%
Proposed	87.72%	91.24%	90.16%	94.4%	93.75%	96.27%	99.73%

**Table 7:** The ablation experiment results in the laboratory dataset.

Dataset	Algorithm	SNR = 0 dB		SNR = 2 dB		SNR = 4 dB		SNR = 6 dB		Clear	
		Avg%	F1%	Avg%	F1%	Avg%	F1%	Avg%	F1%	Avg%	F1%
L1	WDCNN	73.6	71.32	80.25	78.37	84.68	83.36	88.64	88.60	95.20	95.24
	Resnet	84.64	81.40	82.61	80.81	86.11	83.53	91.13	89.61	96.73	96.73
	KD-ResNet-DA	88.01	85.96	88.14	87.06	92.70	91.67	90.20	89.09	96.45	96.37
	Proposed	93.43	93.07	96.17	96.06	96.68	96.60	97.76	97.66	98.80	98.80
L2	WDCNN	72.00	71.43	73.66	72.47	80.30	79.92	84.02	83.15	90.88	88.63
	Resnet	70.01	65.97	78.67	76.50	83.46	82.96	90.90	87.48	97.4	97.35
	KD-ResNet-DA	74.06	73.14	86.17	84.05	88.89	88.43	91.36	90.34	96.60	96.49
	Proposed	88.00	85.74	91.20	91.00	93.88	93.47	94.06	93.78	98.36	98.32
L3	WDCNN	76.58	74.42	82.69	80.22	84.82	82.69	85.68	83.98	90.18	88.28
	Resnet	72.10	70.32	75.67	71.68	80.99	78.09	86.32	84.12	88.82	86.99
	KD-ResNet-DA	80.10	76.72	84.70	83.26	89.50	88.05	91.03	90.12	93.70	93.54
	Proposed	85.68	83.87	95.12	94.66	96.80	96.77	98.87	98.85	98.95	98.91

**Table 8:** Accuracy of laboratory data ablation experiments.

Algorithm	10	16	20	40	60	120	200
S	93.22%	93.53%	93.36%	91.57%	95.84%	98.36%	99.2%
KD	92.96%	94.2%	96.24%	96.24%	98.12%	98.88%	99.34%
DKD	94.87%	95.6%	94.58%	94.65%	98.30%	97.73%	99.2%
CNN-MPDKD	95.2%	96.21%	97.2%	97.27%	98.40%	99.18%	99.67%
Proposed	96.17%	96.6%	98.80%	98.88%	98.93%	99.51%	100%

**Table 9:** Accuracy and F1 score under different signal-to-noise ratios and salt and pepper noise.

SNR	−4 dB	−2 dB	0 dB	2 dB	4 dB	6 dB
Avg%	90.20	92.81	95.21	96.89	97.05	99.12
F1%	89.33	92.04	93.85	96.83	96.97	99.12

## Data Availability

The data used in this study can be requested from the corresponding author. Due to confidentiality requirements in the laboratory where the testing equipment is located, these data are not publicly disclosed.
